# Shrubs indirectly increase desert seedbanks through facilitation of the plant community

**DOI:** 10.1371/journal.pone.0215988

**Published:** 2019-04-24

**Authors:** Alessandro Filazzola, Amanda Rae Liczner, Michael Westphal, Christopher J. Lortie

**Affiliations:** 1 Department of Biology, York University, Toronto, Ontario, Canada; 2 Bureau of Land Management, Central Coast Field Office, Marina, California, United States of America; Shandong University, CHINA

## Abstract

The mechanisms supporting positive ecological interactions are important. Foundation species can structure desert biodiversity by facilitating seedbanks of annual plants, but the direct and indirect mechanisms of shrub effects on seedbank have not been experimentally decoupled. We conducted the first test of shrubs increasing seedbank densities through direct effects on the seedbank (i.e. shrub seed-trapping, animal-mediated dispersal) and indirect effects by facilitating the annual plant community (i.e. seed deposition, annual seed-trapping). Two distinct desert ecosystems were used to contrast transient seedbank densities in shrub and open microsites by manipulating annual plant density and the presence of the persistent seedbank. We measured transient seedbank densities at the end of the growing season by collecting soil samples and extracting seeds from each respective treatment. Transient seedbank densities were greatest in shrub canopies and with relatively higher annual plant densities. The persistent seedbank contributed to transient seedbank densities only in one desert and in the open microsite. Shrubs indirectly increased seedbank densities by facilitation the seed production of the annual plants. Therefore, shrubs are increasing seedbank independently of the annual plant community, likely through trapping effects, and dependently by facilitating seed production of the annuals. These findings provide evidence for a previously undescribed mechanism that supports annual seedbanks and thus desert biodiversity. We also identify shrubs as being significant drivers of desert plant communities and emphasize the need to consider multiple mechanisms to improve our ability to predict the response of ecosystems to change.

## Introduction

Seedbanks contain the biodiversity of annual plants in deserts, but their density is variable. Different subsets of plant species germinate in response to precipitation variation with the remaining species present within the seedbank [1,2]. Mechanisms that maintain and contribute to desert seedbanks are thus responsible for the long-term persistence of species within the annual community. These mechanisms can be classified into seed production, seed persistence (i.e. granivory or decomposition), and seed dispersal [3–5]. Understanding the mechanisms that contribute to seedbank densities can improve our ability to predict how desert communities will respond to change. However, previous research exploring the mechanisms that contribute to seedbanks has been generally focused on climate patterns [6], disturbance [7], or animal interactions [8]. Dominant plants can also play a dominant role in structuring seedbanks, although these relationships are relatively understudied [9]. Dominant plants in deserts, such as shrubs, can increase the density of seedbanks within the canopy relative to open spaces through trapping dispersing seeds [3,5,10]. Exploring the mechanisms that shrubs contribute to seedbanks is important because it could be a significant driver that maintains desert biodiversity.

In deserts, shrubs can act as foundation species that increase seedbank densities by directly trapping seeds or by indirect mechanisms through an intermediary animal or plant species [3,5]. Shrubs significantly influence the movement wind or water around their canopy [11] and thus can directly trap seeds or act as barrier for movement [5]. Shrubs can also have indirect effects that can be separated into those with an animal or plant as intermediary. For instance, shrubs can indirectly facilitate seed arrival by acting as a perching site for seed-carrying birds [12] or as cache for granivorous rodents [4]. Additionally, shrubs can indirectly increase seedbank by facilitating the annual plant community that increases seed production [13] or increase seed trapping rates [14]. The size of a shrub can also impact the composition of annual plants [15] or the arrival of seed [13] because larger shrubs can physically obstruct more seeds and provide greater facilitative effects. Larger shrubs can intercept more solar radiation [15], have higher soil nutrients [16], or lower evapotranspiration [17] creating a favourable microclimate for desert annuals. Previous research has explored indirect shrub-seedbank effects with animals [3,12], but to our knowledge, there have been no studies that identified indirect facilitation with the annual community. Indirect shrub-seedbank effects need to be measured because they can be more sensitive to environmental change due to their reliance on the presence of multiple species and the interactions among them.

The purpose of this study was to examine the mechanisms that shrubs increase seedbanks by experimental manipulation in two desert ecosystems. We chose to assess the indirect shrub-seedbank effects with plants from other shrub effects on seed arrival as this is the initial test of these mechanisms. In the Mojave and San Joaquin Deserts, we manipulated annual cover and seedbank presence to determine effects of shrubs on seedbank. We hypothesized that shrubs increase seedbank densities by indirectly facilitating the annual plant community that collectively increases both seed production and trapping. We predicted that (i) shrubs directly increase the density of the annual plants and the effect is proportionate to the shrub’s size, (ii) shrubs increase seedbank densities independent of the annual plant community that is also proportionate to the shrub’s size, and (iii) shrubs indirectly increase seedbanks densities by facilitating the annual plants, which increase seed deposition and the trapping effects by the annuals themselves.

## Methods

### Study site

In California, U.S.A., we selected three sites within the Panoche Hills Ecological Reserve in the San Joaquin Desert [18] and three within the Mojave National Preserve in the Mojave Desert (S1 Appendix). The Mojave National Preserve has mean annual precipitation between 34 and 310 mm per year [19] and mean monthly temperatures of 8.61 °C in January and 33.9 °C in July as recorded at Zzyzx, California (National Park Service, latitude: 35.14, longitude: -116.12). The growing season for annuals in the Mojave Desert is typically from November to April [20]. The soil composition of the Mojave sites were characteristic of shrublands dominated by *Larrea tridentata* (Creosote bush) with a sandy substrate: sand = 78.7%, silt = 14.9%, and clay = 6.4% (Lei 1998). Panoche Hills has mean annual precipitation of 229 mm [21] and mean monthly temperatures of 8.9 °C in January and 26.1°C in July as recorded at Los Banos, California (37.05, -120.85; http://www.usclimatedata.com/). The growing season for annuals in the San Joaquin Desert is also typically from November to April [21]. Soils composition at Panoche Hills is a shrubland dominated by *E*. *californica* and has soil that is well-drained and varies from sandy loam to gravely loam based on slope [22]. These two deserts were chosen because they have similar seasonality (i.e. arid rain-shadow deserts with the same growing season), but distinctly different composition of plant communities. The intention was to compare if the trends in our results are similar among other plant assemblages with similar climate patterns. Panoche Hills was tested in both 2013 and 2014. However in 2014, California experienced the most severe drought within the last 100 years [23], and this region was particularly impacted (Figure C in S1 Appendix). Consequently, the Mojave Preserve was only tested in 2013 because there was exceptionally low plant germination in 2014. Permits were obtained from the National Park Service for the Mojave National Preserve (MOJA-00279) and from Bureau of Land Management for Panoche Hills (Central California Field Office).

### Species descriptions

The dominant shrubs used in this study were *Ephedra californica* (Ephedraceae) in Panoche Hills and *Larrea tridentata* (Zygophyllaceae) in the Mojave Preserve. The dimensions of each shrub individual examined were measured with diameter 1 (D1) being the length of the longest side of the shrub, diameter 2 (D2) the length of the shrub perpendicular to D1, and the height (H) from the base of the basal root to the tallest branch. The volume of the shrub was calculated using a modified version of the equation for a semi-sphere (Eq 1) [24]. In Panoche Hills, the average shrub density of *E*. *californica* was approximately 0.43 individuals per 100 m^2^ [24] and the volume ranged between 0.41 and 14.0 m^3^ (mean ± SE = 4.01 ± 0.19 m^3^). In Mojave Preserve, the density of *L*. *tridentata* was similar to other areas within the preserve between 2–4 individuals per 100 m^2^ [25] and the volume ranged between 0.29 and 4.67 m^3^ (mean ± SE = 1.74 ± 0.12 m^3^). Panoche Hills is heavily invaded by non-native plants that includes the annual forbs *Bromus madritensis ssp*. *rubens*, *Erodium cicutarium* and *Schismus barbatus* (S2 Appendix). The Mojave Preserve is less invaded, but non-native species such as *B*. *madritensis*, *E*. *cicutarium* and *S*. *barbatus* are still common (S2 Appendix). For Panoche Hills and Mojave Preserve, a full list of the species observed and the proportion of non-natives present can be found in S2 Appendix.

$$Volume=\frac{2}{3}\times \pi (\frac{D1}{2}\times \frac{D2}{2}\times H)$$ (1)

### Annual and seedbank manipulations

To test the mechanisms of seed arrival we applied four treatments orthogonally to 71 cm^2^ plots manipulating annual plants (P) and the persistent seedbank (S) (Table 1). We divided the seedbank as ‘transient’ after all seeds had fallen by the annual plant community (i.e. May) and ‘persistent’ for the ungerminated seed after the rainy season (i.e. February). We selected ten shrub-open pairs at each site and deployed the plots in a randomized linear array that paralleled the shrub dripline with a 5 cm buffer between treatments (S3 Appendix). The pots were placed in a randomized linear array to ensure each were equal distant to the center of the shrub because distance from the shrub’s base stem effected seed disposition (S1 Fig.). The shrub plots were placed under canopy dripline to maximize the seed capture effect (S1 Fig.) and on the north-facing side because this side has the greatest facilitation effect in the northern hemisphere. The open plots were placed two meters away from each shrub, but in the same area as the paired shrub treatment. We assumed that each treatment within a shrub or open microsite experienced similar seed dispersal that was mediated by the presence of annual plants and supplemented by either annual deposition by the plants within the treatment or the persistent seedbank if left intact.

**Table 1:** A list of the mechanisms that contribute to transient seedbank densities and the method of calculation for the effect sizes based on the differences between treatments. The treatment plots that were used measured in shrub and open microsites included +P+S, +P-S, -P+S, & -P-S.

Effect estimate	mechanism	treatment	control
Trapping by annual plants	Physical obstruction of seeds	+P -S	-P -S
Ambient community	Annual trapping and seed production/deposition	+P +S	-P +S
Seedbank carry over	Persistent seedbank after emergence, seed decomposition, and granivory	-P +S	-P -S
Other shrub effects	Trapping or animal-mediated dispersal of seeds	shrub -P -S	open -P -S

Each treatment was two-level that included the presence or the entire removal of plants or seedbanks (+P+S, -P +S, -P -S, & +P–S; S4 Appendix). The treatment plots were 9.5 cm diameter pots that were 12 cm in height and were buried so that the lip of the pot was flush with the adjacent soil. The pots were cylindrical that narrowed towards the base with an approximate soil volume of 700 cm^3^. These pots were used to prevent mixing of the natural soil with the soil in the treatments. In all treatments, the soil and ambient plants were carefully placed into the pots so to not disturb the natural plant cover or soil surface texture. The -P treatments were applied by removing (i.e. manually pulling) all live plant material within the treatment plot. The–S treatments were applied by sieving all the soil in the treatment plot using a 500 micron sieve, removing seeds or organic material that was caught by the sieve, and then returning the sieved soil into the treatment plot. The +P-S treatment used ten artificial annuals made from bamboo pieces to replicate the seed trapping effects of annual plants. These ten pieces kept standard for all sites and the number was chosen *a priori* to reflect a moderately dense clump of annuals for both deserts. This mimic was necessary to have a fixed density of plants in a plot where the soil was disturbed and functioned as an approximation of the trapping effect by annual plants without the seed deposition of a naturally occurring annual. The treatments were applied during the growing season in February after germination and prior to new seed deposition. The total number of samples collected overall (N) was 720 with 10 replicates (*n*) of each treatment (Mojave National Preserve = 240; Panoche Hills = 480). Full dataset available online at (https://knb.ecoinformatics.org/view/doi:10.5063/F17H1GWM).

On April 28^th^ of 2013 and 2014 following seed set by annual plants, the soil from the top 10 cm of each treatment was collected and dried at 80° C in a mechanical convection oven for three days. The soil was then sieved using a 500 micron sieve to extract seeds for counting. The dried soil was also weighed to standardize the number of seeds per gram of soil. Therefore, the number of seeds divided by the weight of the dried soil in grams (hereafter *seed*.*soil*) was used as the response variable for the transient seedbank. We also measured annual plant density and species richness in a 50 x 50 cm quadrat adjacent to the four treatment plots.

To ensure our method of collecting seeds was effective, we placed 100 soil samples of each Mojave Preserve 2013, Panoche Hills 2013, and Panoche Hills 2014 into germinating conditions. These soil samples were put into 10 cm^2^ pots, placed into a greenhouse, and watered daily for two weeks. Eleven plants had emerged from the 300 samples in the greenhouse suggesting that the percent chance one seed capable of germination was missed during extraction per sample was approximately 3.6%. However, the average number of seeds extracted in the soil samples were 11.24 suggesting the overall volume of seeds that were missed was approximately 0.32%.

### Data analyses

We tested for differences in the annual plant community between shrub and open microsites by fitting a generalized linear model with annual plant density and richness as the response variables and microsite and desert site used as factors. The models were fitted with a negative binomial error distribution using *glm*.*nb* function, package *MASS* [26] in *R* version 3.4.3 [27] because plant density and species richness both represented a discrete count that is over-dispersed [28]. Post-hoc analyses were conducted using least-square means with Bonferroni correction for multiple comparisons using *lsmeans* function, packag*e lsmeans* [29].

We statistically contrasted shrub-seedbank effects on *seed*.*soil* using linear mixed models *lmer* function, package *lmerTest* [30]. We fitted fully factorial models with microsite, presence of annuals, and presence of persistent seedbank as predictor variables. We square-root transformed *seed*.*soil* to meet assumptions of normality for all analyses. Linear mixed model were fitted separately for Mojave Preserve and Panoche Hills because of different shrub species that were tested and different number of years that the experiment was conducted. Local site was fitted as a random effect in both models, and year was fitted as a random effect only in the Panoche Hills model. We then used F-tests with degrees of freedom calculated using the Satterthwaite approximation [31] to determine *seed*.*soil* significance in both models [31]. To determine if shrub volume effects seedbank densities independent on the annual community, we first removed the effects of the annual plant densities on the seedbank. We did this by fitting a separate linear mixed model with *seed*.*soil* as the response variable, no intercept, and annual plant density as the predictor variable. The resulting residuals represented relative seedbank densities after accounting for variation due to annual plant densities. We then fitted the residuals against a model with shrub volume as the predictor and the random effects local site and year (Panoche Hills only).

To determine sign and magnitude of the mechanisms that contributed to increasing seedbank densities (Table 1), we used bootstrapped effect estimates [32]. The effect sizes were determined using a linearly weighted combination of means (*bootES* function, package *bootES*) where values of +1 represent treatment effect and -1 a control effect [32]. Bootstrap methods approximate the unknown distribution of the effect sizes by resampling the original data with replacement (2000 iterations) and contain the same number of data points as the original sample [32]. Confidence intervals were calculated using the bias-corrected and accelerated bootstrap method [33]. The effects of the mechanisms were calculated between plots with differences in presence of annuals or persistent seedbank (Table 1). We compared effect sizes between shrub and open microsites using t-tests on the bootstrapped estimates of each mechanism.

## Results

### Direct effects of shrubs on annuals

**Fig 1:**
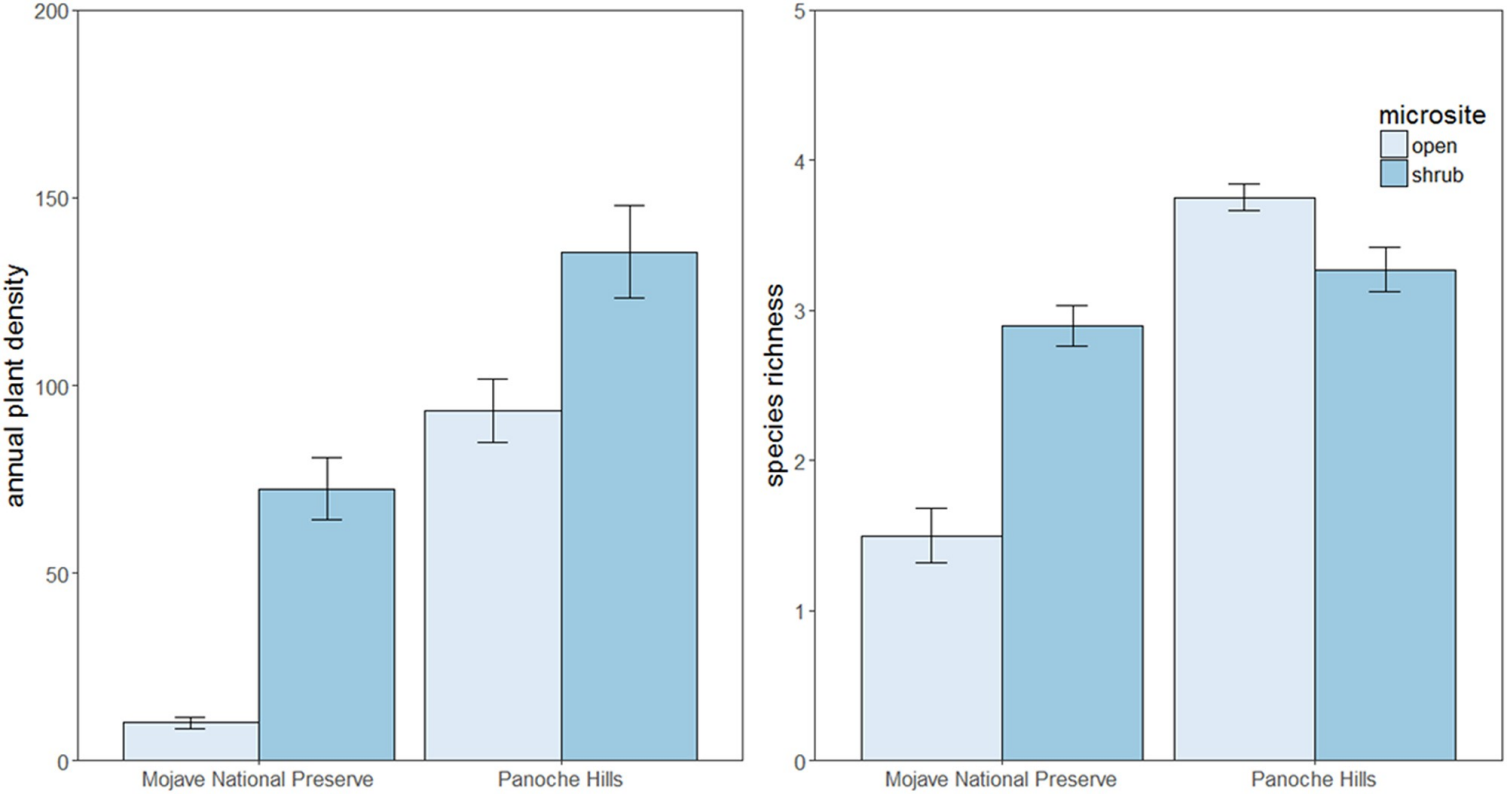
The annual plant density and annual species richness for shrub and open plots in the Mojave National Preserve and the Panoche Hills in both years. Each bar represents the mean with standard error.

Plant densities were significantly greater in shrub microsites (χ^2^ = 95.2, p < 0.001; Fig 1) and at Panoche Hills (χ^2^ = 414.9, p < 0.001; Fig 1). In the Mojave Preserve, shrubs had significantly greater annual plant density and species richness than in Panoche Hills (post-hoc contrasts p < 0.001; Fig 1). In Mojave Preserve, annual plant density was greater under shrub canopies, but species richness was significantly greater in open microsites (post-hoc contrasts p < 0.001; Fig 1). Greater shrub volumes increased annual plant density at both Mojave Preserve (mean effect = 0.81, SE = ± 0.072, p < 0.001) and Panoche Hills (mean effect = 0.051, SE = ± 0.011, p < 0.001).

### Direct and indirect effects of shrubs on seedbank

**Fig 2:**
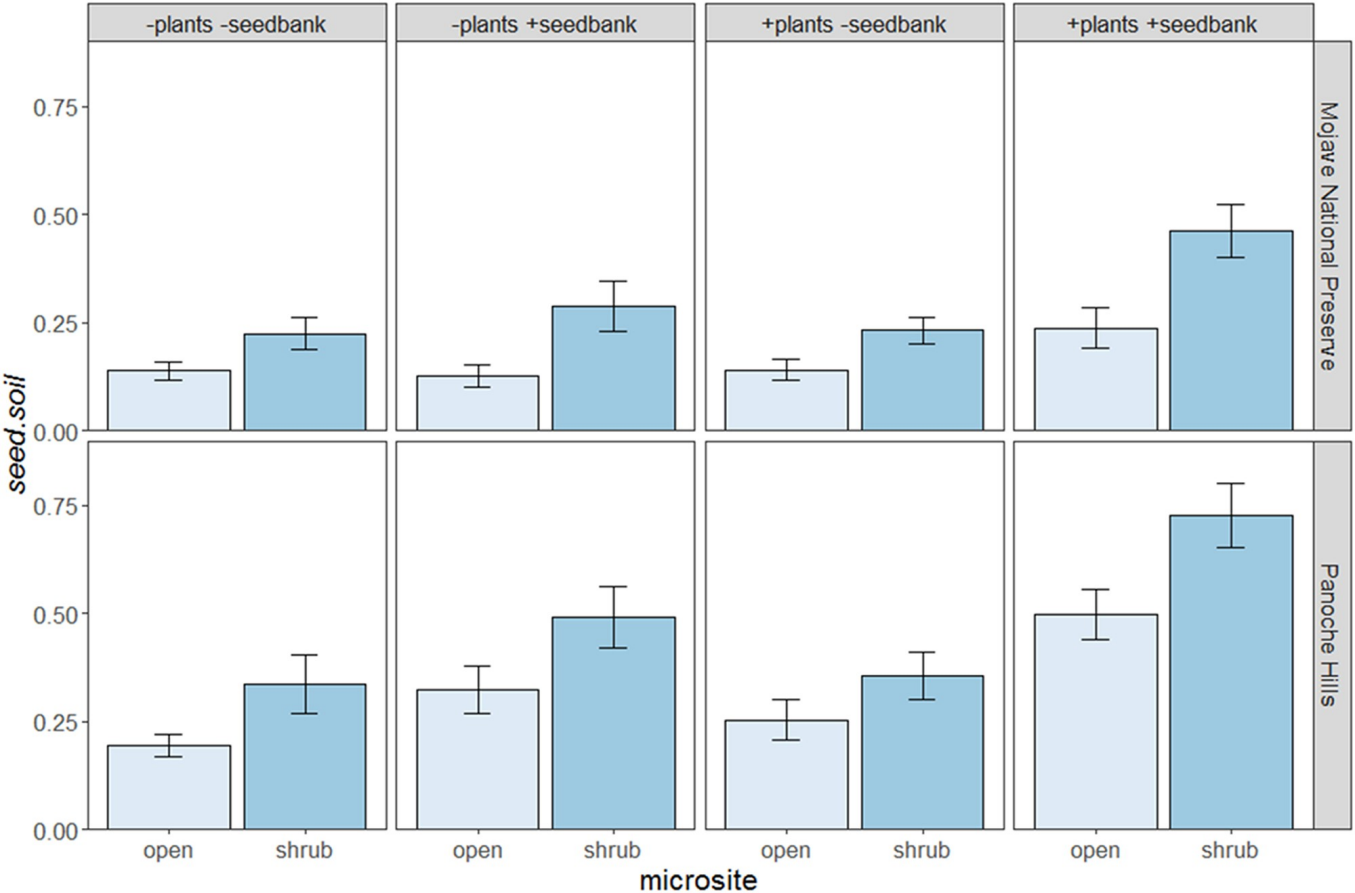
The differences in seed.soil for each of the four treatments and in each microsite (shrub and open) for the Mojave National Preserve (MNP) and the Panoche Hills (PAN). Each bar represents the mean with standard error.

**Fig 3:**
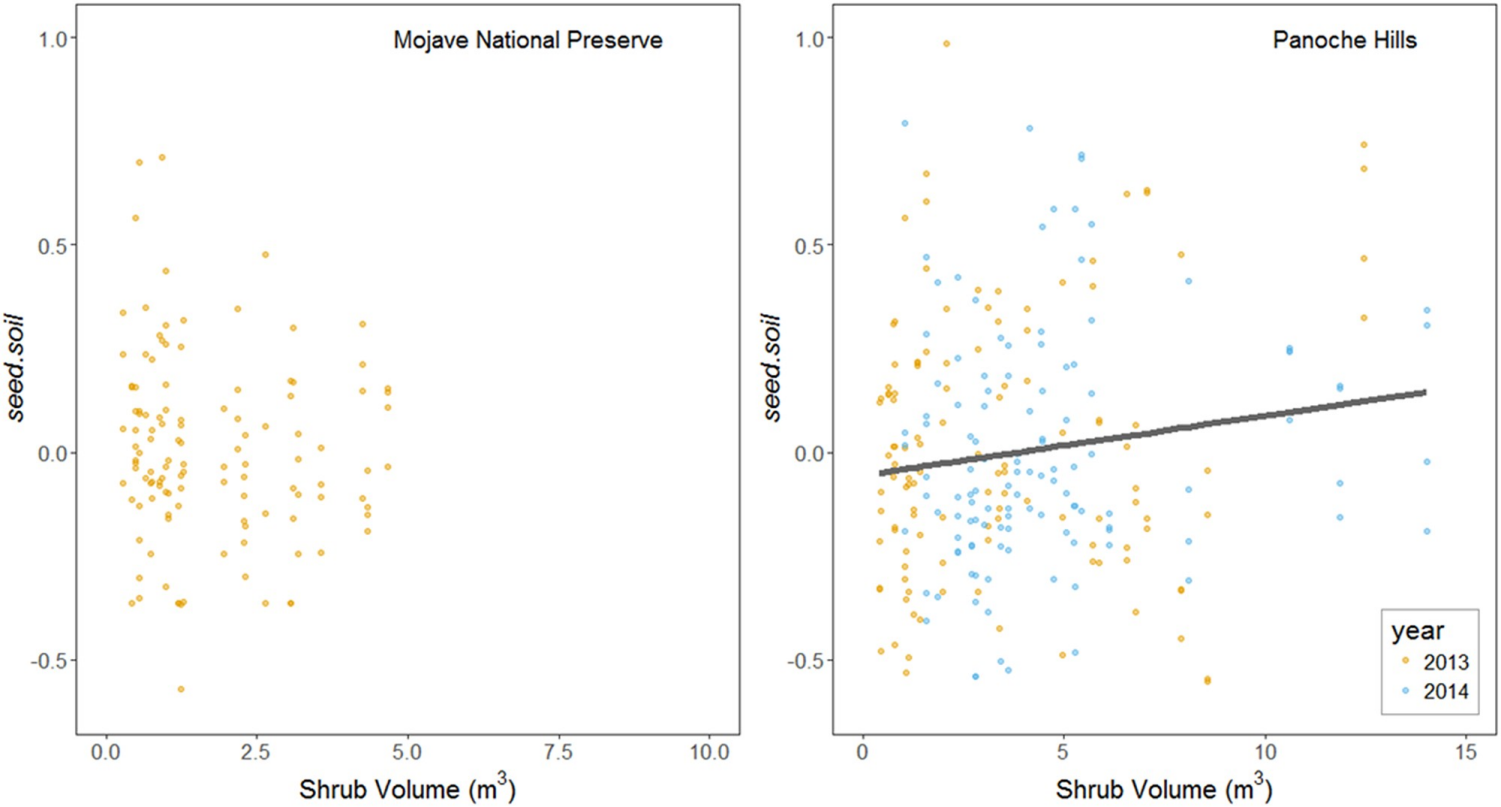
Transient seedbank (*seed*.*soil*) densities increase the size of the shrub at Panoche Hills, but not the Mojave National Preserve. The solid line is mean model fit at mean of *seed*.*soil* residuals, i.e. after compensating for effect of annual plant densities on seedbank densities. (Panoche Hills: R^2^ = 0.20).

**Fig 4:**
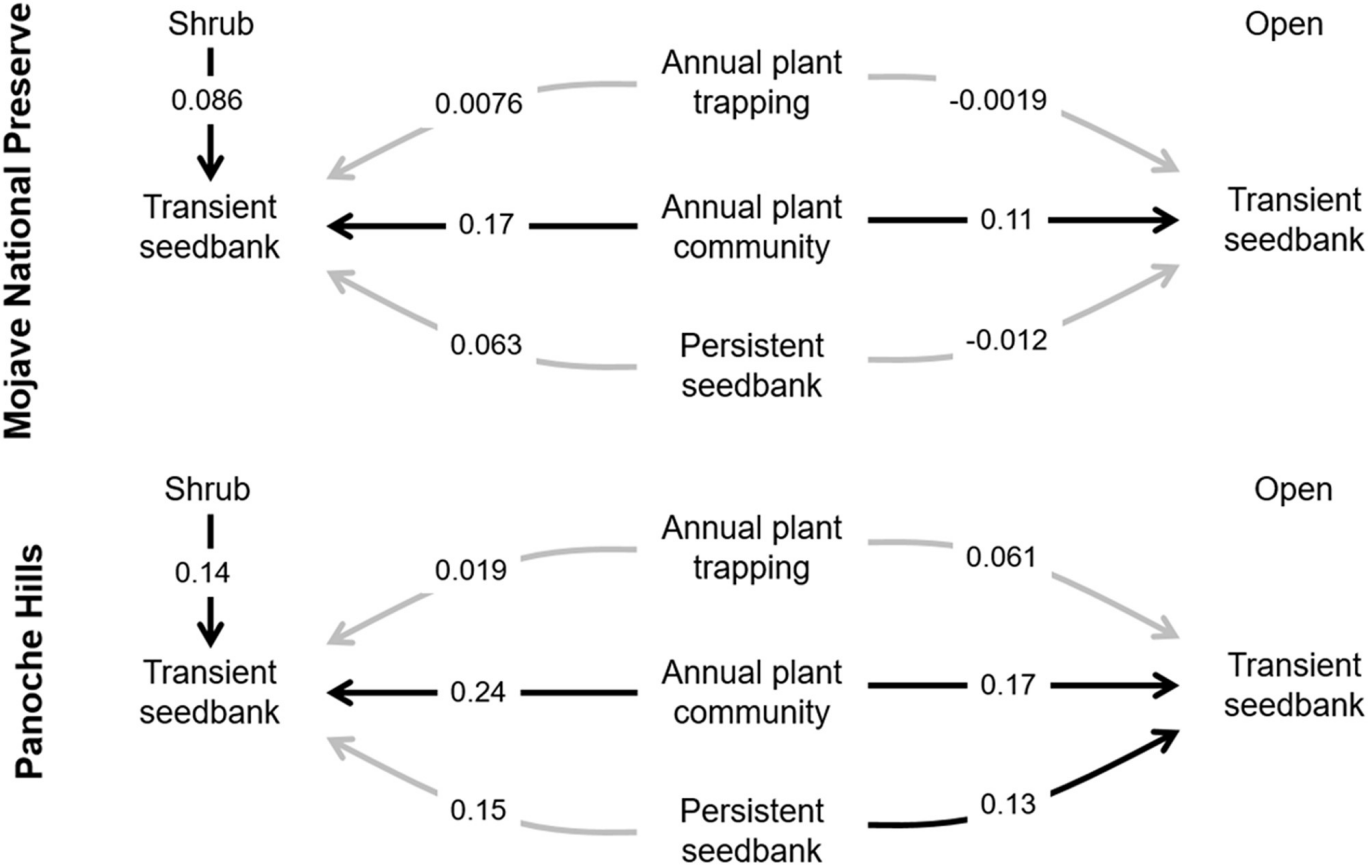
The effect sizes of each mechanistic process in the shrub and open microsites that contribute to the transient seedbank (*seed*.*soil*). The shrub arrow represents trapping effect only. Sites represented are the Mojave National Preserve in 2013 and Panoche Hills both in 2013 and 2014. Black arrows show significant effects (α < 0.05) based on confidence intervals calculated using the adjusted bootstrapped percentile (BCa) method and grey arrows represents non-significance.

In both deserts, shrubs significantly increased densities of the transient seedbank (Table 2), but not the persistent seedbank (Table 2, Fig 2). Shrubs with greater volume significantly increased seedbanks independent of annual plant densities in the plots at Panoche Hills (F_238_ = 4.94; p = 0.027; Fig 3) but not at Mojave National Preserve (F_118_ = 0.92; p = 0.34; Fig 3). The ambient, annual plant community consistently contributed to the transient seedbank through seed deposition in both microsites and both deserts (Table 2, Fig 4). There was no shrub by annual interaction effects on the transient seedbank (Table 2). The independent effect of the ambient annual plant community—excluding trapping—was significantly greater in shrub microsites relative to open (p < 0.001; Table 1, Fig 4). The annual trapping effect estimated using the artificial mimics did not have any significant effect on the transient seedbank densities (Fig 4). The persistent seedbank did not significantly contribute to the transient seedbank in Mojave Preserve or the shrub microsite in Panoche Hills, but was a significant contributor in the open microsite in Panoche Hills.

**Table 2:** Linear mixed models testing for differences in *seed*.*soil* among microsites (shrub/open) and each of the treatments that manipulated either annual plant density or the persistent seedbank. The annual plant density of neighbouring annuals was included as a covariate. Significance at α < 0.05 is denoted by bolded values. The sign of significant effects were positive.

Treatment	DF	F-value	p-value	DF	F-value	p-value
	*Mojave National Preserve*			*Panoche Hills*		
microsite	465	16.7	**<0.001**	465	5.86	**0.016**
annual plant density	134	6.47	**0.012**	134	19.1	**<0.001**
persistent seedbank	468	7.06	**0.0084**	468	57.7	**<0.001**
microsite * annuals	468	0.012	0.92	468	0.095	0.76
microsite * persistent seedbank	468	1.63	0.20	468	0.36	0.55
annuals * persistent seedbank	468	6.19	**0.013**	468	5.58	**0.018**

## Discussion

Positive interactions are important for structuring the communities of desert plants [34]. Here, shrubs consistently increased seedbank densities both dependently and independently of facilitation effects on the annual plant community. Our predictions that shrubs increase seedbank densities and increased annual seed deposition were supported. The density of annual plants was also related to increased seedbank densities in both shrub and open plots. Indirect shrub facilitation by annual plants trapping seeds was not a significant effect that contributed to transient seedbank densities, but this could be due to the artificial annuals that did not effectively mimic the seed trapping effect of annual plants. The persistent seedbank did not significantly contribute to the transient seedbank in either desert or microsite except the open microsite in Panoche Hills. Within our study, shrubs did not increase the persistent seedbank in either desert system and thus were not likely increasing the temporal heterogeneity of seedbanks. Instead, these findings support that persistent seedbank densities were similar throughout time and that the supply was balanced with the consumption of the persistent seedbank. Trapping by shrubs, but not annuals, and indirect facilitation by shrubs on annual plant reproduction are dominant mechanisms that increase densities and spatial heterogeneity of seedbanks.

### Shrub effects on seedbank

Density and richness are commonly reported responses in the facilitation literature, but both are not necessarily influenced simultaneously in a given system. Here, shrubs increased the density of seedbanks and the annual plants but not total species richness. Positive interactions are largely species specific that provide a favourable microclimate for select species and not others [35]. The shrubs within our study reduced microclimatic variation that can benefit more competitive species and exclude those that are stress-tolerant [21,35,36]. The facilitation effect was greatest in the Mojave Desert relative to the San Joaquin Desert and this could have been driven by greater differences in microclimates and a higher proportion of exotics in Panoche Hills. Each desert represented unique plant assemblages, and the target shrub species were different in traits. *L*. *tridentata* in the Mojave Desert is smaller and less dense than *E*. *californica* (S2 Fig), but interestingly direct shrub effects on seedbank was the dominant mechanism in both systems. Instead of physically obstructing seeds, *L*. *tridentata* could be increasing seedbank through animal-mediated dispersal, such as acting as perch site for birds [37] or rodent cache [4] which are independent of shrub size. Alternatively, *E*. *californica* could have a significantly higher rate of seed removal relative to *L*. *tridentata* by providing cover for granivorous animals [38,39]. The effects of these shrubs on animal-mediated dispersal are largely unknown and an opportunity for future research to better untangle the facilitation effects on seedbank.

Transient and persistent seedbanks have different ecological implications. Persistent seedbanks provide resilience to ecosystems with high climatic variability and support the long-term biodiversity of annual plant species [40]. However, we found that shrubs significantly influence the transient but not persistent seedbank. There was also a high frequency of invasive species, such as *Bromus spp*., that do not form persistent seedbanks [41]. For instance, the main predictor of seedbank densities for *Bromus tectorum* in the Great Basin Desert has been shown to be precipitation and deposited seeds rarely persist beyond a second year in the soil [42]. The favourable microclimate conditions for some species, but not all species, within the shrub also encourages germination and increases the ratio of emerged plants to persistent seedbank densities [1,43]. As a result, the open microsites can be more dependent on the persistent seedbank because of higher climatic variability [44], as detected within this study for the Mojave Desert. Although not tested here, the type and quality of seeds between microsites can also be different. Shrubs tend to have larger seeds present in the seedbank because the open can only retain small seeds with diaspores that are trapped on the surface of the bare ground [45,46]. The shrub microclimate is also often cooler with less temperature extremes and higher humidity that can increase the longevity of viable seeds in the persistent seedbank [47]. For instance, the annual *Salvia columbariae* is present at both desert sites and has reportedly higher rates of germination when stored at temperatures below 20°C and with high humidity [48]. This potential difference is largely consistent with ecological theories and empirical research that suggest seed dormancy increases with aridity and climate variability [49–51] suggesting the Mojave Desert is more dependent on temporal storage and the persistent seedbank. Seedbanks within shrub canopies can be less reliant on persistent seedbanks thereby making them sensitive to disturbances that threaten these positive interactions.

### Indirect shrub effects on seedbank

Shrub effects on seedbanks can be complex and involve indirect interactions with annual plant communities. The mechanism that the annuals contributed to seedbank densities appears to be through increased deposition or seed rain by the annuals and not by direct seed trapping by the shrub structure. A common dispersal strategy for desert annuals is atelechory, where seeds travel short-distances relative to the parent plant because long-distance dispersal often does not provide a benefit in an environment that is variable and resource limited [52]. Similarly, we also observed higher seedbank densities associated with high annual densities and in shrub canopies. Desert shrubs can also increase seed production [53], and this effect can be a relevant mechanism here contributing to the observed differences in the seedbank. For instance, competitive plant species, such as *Bromus spp*. and *Erodium spp*. deposit significantly higher seeds in shrub canopies relative to open spaces [53,54]. Shrub facilitation are thus increasing annual plant densities, but also likely the production of seeds. Decoupling the different pathways that shrubs contribute to seedbanks is necessary for understanding the spatial distributions of desert plant communities.

### Shrub effects on annual life-stage

Shrub facilitation of other plants can be life-stage specific and is greatest in early plant life-stages [43,55]. This experiment supports previous research that shrubs facilitate annual plant communities by increasing seed production due to higher plant densities and seed arrival [3,5]. Seeds under shrub canopies can also have a higher probability of emergence and establishment relative to open spaces [56]. This shrub-based facilitation on seed germination is species specific because of the favourable microclimatic conditions and not because of ecotypic associations in the annual populations with the shrub microsite [57]. However, the annual plant species can have seeds evolved to be trapped in shrub canopies to increase the probability of favourable germination conditions through specific morphological adaptations [58]. As the growing seasons continues, previous research has shown that facilitation can shift to competition in deserts as resources become less available [43,55]. For instance, there can be a mismatch in the microscale distribution of plants and their respective seedbank with higher seed densities within shrub canopies but higher annual densities outside the shrub [10]. This is the concept of negative density-dependence where communities that have initially higher densities experience greater mortality [59]. However, our findings suggest that the overall effect of shrubs on the annual species is net positive because the increases in seedbank densities and plant establishment are not limited by competition in later life-stages. At the end of the growing season, we observed either a positive or neutral effect of shrubs on plant density and seed production in both deserts. This is an example of positive density-dependence that is more typical in environments with high abiotic stress [60]. To better advance community theory, it is necessary to examine net outcomes through different measures of plant performance [61] and in different microsites such as shrub-open.

### Implications

Shrub facilitation is a critical component of community resilience by supporting the emerged annual community and increasing its seedbank. In desert ecosystems, shrubs aggregate resources, seedbanks, local biodiversity, and can enhance net productivity [62]. Additionally, we found initial evidence of the indirect effects of shrubs on desert seedbank through facilitation of the annual plant community and confirm that this is a relevant pathway that increases seedbank. We have identified dominant plants as a significant factor mediating seedbank dynamics through a series of direct and indirect mechanisms. Shrubs can also affect seed viability because of differences in microclimate [47] and mediate seed dispersal by animals [4,37], but we did not explore these mechanisms. Future research should empirically test shrub effects on animal-mediated dispersal, seed viability, and the mechanisms within this study to construct a structural equation model that better quantifies the direct and indirect effects of shrubs on seedbanks. Each of these mechanisms contribution to seedbank densities likely responds independently to changes in climate patterns, disturbance, or other factors. This specific form of spatial heterogeneity relative to open microsites within a region suggests that shrubs can be sources for plants species that are recruitment limited—i.e. source populations [63,64]. For example, seedbanks can buffer against extreme climate events, such as drought [65]. However future environmental extremes could surpass thresholds that then significantly impact community structure [66,67]. Recent climate projections suggest that greater periods of drought in California could surpass previous thresholds [23] and can increase shrub mortality [68]. Shrubs are also often impacted by land use practices in deserts, such as agriculture, grazing, and solar farm development [69,70]. Future land management must consider the vitality and function of shrubs including their impacts on the seedbank because both direct and indirect effects on biodiversity are complex and critical to resilience in these systems.

## Supporting information

S1 Appendix Location and climate of study sites. (DOCX)

S2 Appendix Identities of annual plants at study sites. (DOCX)

S3 Appendix Images of treatment set up. (DOCX)

S4 Appendix The different treatments used within the experiment. (DOCX)

S1 Fig The seed bank densities per gram of soil for five distances between the shrub center and an adjacent open microsite. (TIF)

S2 Fig Typical examples of the shrubs Larrea tridentata and Ephedra californica in the Mojave National Preserve (Mojave Desert) and Panoche Hills (San Joaquin Desert) respectively. (TIF)
