# Supplementary material for: Shrubs indirectly increase desert seedbanks through facilitation of the plant community
Source: PLoS One. 2019 Apr 24;14(4):e0215988. doi: 10.1371/journal.pone.0215988 (PMC6481865; doi:10.1371/journal.pone.0215988)
Supplement: S1 Appendix — (DOCX) [file pone.0215988.s001.docx]

**Supplementary 1 - Location and climate of study sites**

**Table A:** Location and characteristics of the study sites in the San Joaquin and Mojave Deserts of California, U.S.A.

| Location | Shrub species | Latitude | Longitude | Elevation (m) | Aspect |
| --- | --- | --- | --- | --- | --- |
| Panoche Hills | *Ephedra californica* | 120°47.886'W | 36°41.776'N | 661 | flat |
| Panoche Hills | *Ephedra californica* | 120°48.777'W | 36°42.463'N | 611 | south facing |
| Panoche Hills | *Ephedra californica* | 120°48.862'W | 36°42.336'N | 596 | north facing |
| Mojave National Preserve | *Larrea tridentata* | 115°51.601'W | 35°11.057'N | 737 | flat |
| Mojave National Preserve | *Larrea tridentata* | 115°47.42'W | 35°9.31'N | 951 | flat |
| Mojave National Preserve | *Larrea tridentata* | 115°43.717'W | 35°8.672'N | 1128 | flat |


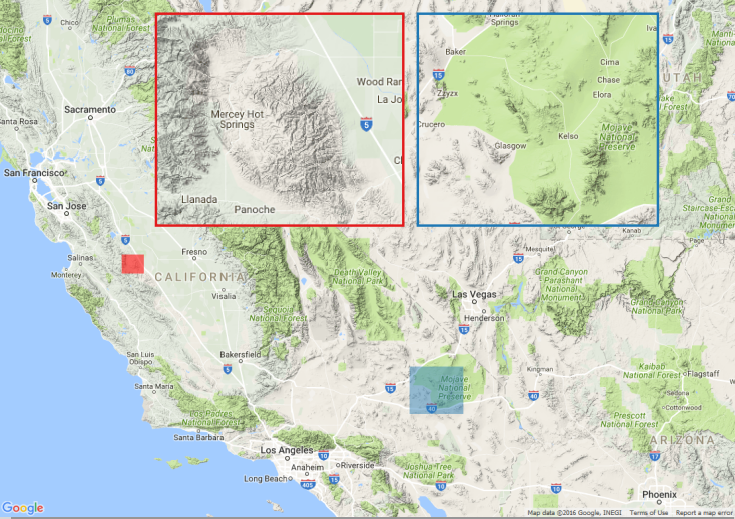


**Figure A:** Location of the Panoche Hills study area (left) in the San Joaquin Desert and the Mojave National Preserve (right) in the Mojave Desert.

Panoche Hills

Mojave National Preserve


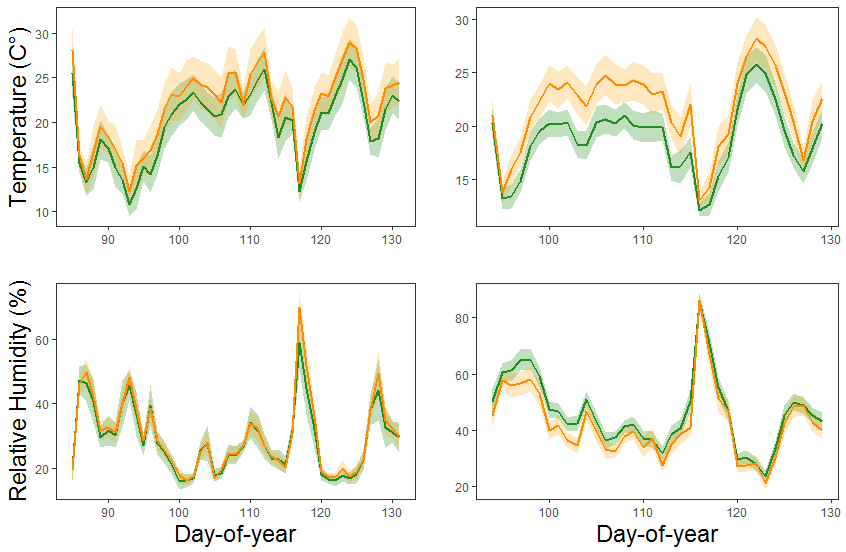


**Figure B:** Temperature and relative humidity of the shrub (green) and open (orange) microclimates in Panoche Hills and Mojave Desert. At each site, a pair of HOBO ProV2 were placed in a shrub and open microsite to measure temperature and relative humidity hourly from March 26^th^ to May 9^th^ 2014. Lines represent means between loggers at the sites within the respective deserts and error bars are 95% confidence intervals.


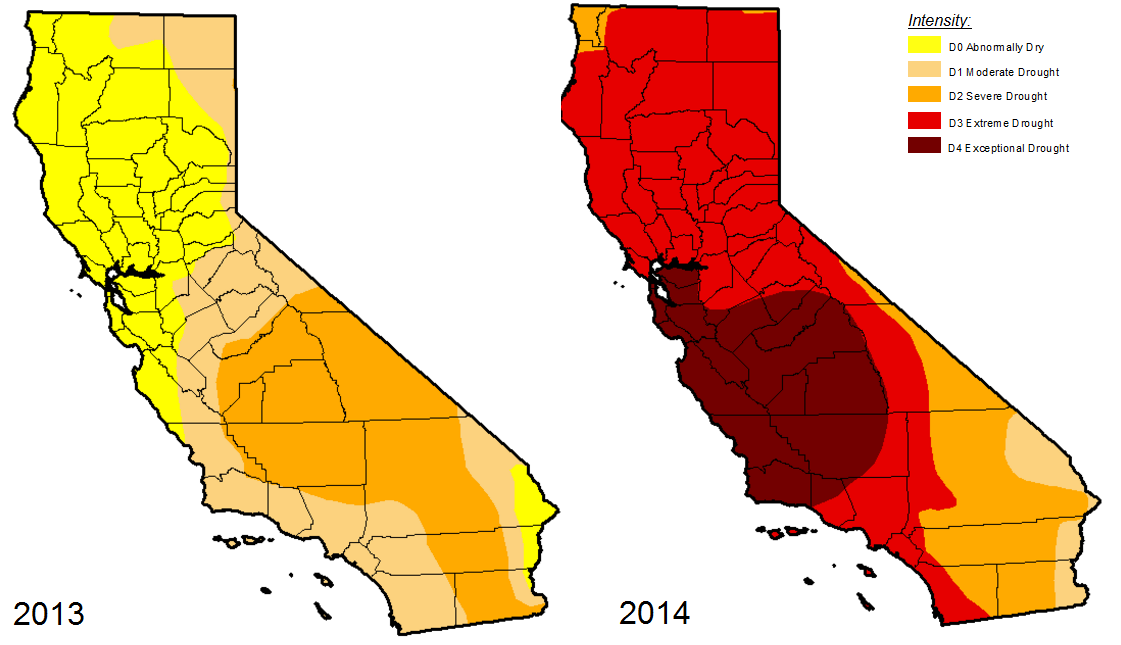


**Figure C:** The drought conditions experienced throughout California on April 28th 2013 and April 30th 2014 respectively based on the United States Drought Monitory by the National Drought Mitigation Center (DroughtMonitor.unl.edu).
