# Supplementary material for: Shrubs indirectly increase desert seedbanks through facilitation of the plant community
Source: PLoS One. 2019 Apr 24;14(4):e0215988. doi: 10.1371/journal.pone.0215988 (PMC6481865; doi:10.1371/journal.pone.0215988)
Supplement: S2 Appendix — (DOCX) [file pone.0215988.s002.docx]

**Supplementary 2 - Identities of annual plants at study sites.**

**Plant surveys in 2015**

In April of 2015, plant surveys were conducted at each of the three sites within MNP and PAN. At each site, 50 x 50 cm quadrats were placed in 15 shrub and open pairs. The quadrats were placed on the north end of the shrub and in the open (greater than 1.5 meters away from any shrub). Individual plants in each quadrat were identified to species level and relative abundances were counted. The conservation status of each identified species (i.e. native or non-native) was determined using the CalFlora database (<http://www.calflora.org/>). The average number of plants surveyed in each desert and microsite were summarized based on the conservation status (Table B.1). A full list of all the species identified at the sites is listed in Table B.2

**Table A:** The average density of annual plants based on species origin in shrub and open plots for each the Mojave National Preserve (MNP) and Panoche Hills (PAN).

| **Site** | **Conservation Status** | **Microsite** | **Density (individuals / 0.25 m^2^)** |
| --- | --- | --- | --- |
| MNP | Non-native | Open | 7.80 |
| MNP | Non-native | Shrub | 61.47 |
| MNP | Native | Open | 18.60 |
| MNP | Native | Shrub | 30.67 |
| PAN | Non-native | Open | 78.53 |
| PAN | Non-native | Shrub | 134.40 |
| PAN | Native | Open | 10.40 |
| PAN | Native | Shrub | 3.80 |

**Table B:** List of annual species observed at the study sites in the Mojave National Preserve and Panoche Hills.

| **Site** | **genus** | **species** | **species.name** | **sub.spp** | **common.name** | **Life-form** | **status** |
| --- | --- | --- | --- | --- | --- | --- | --- |
| MNP | *Bromus* | *hordeaceus* | *B.hordeaceus* | | soft brome | grass | exotic |
| MNP | *Bromus* | *madritensis spp. rubens* | *B.rubens* | | red brome | grass | exotic |
| MNP | *Cryptantha* | *barbigera* | *C.barbigera* | | cryptantha | herb | native |
| MNP | *Camissonia* | *brevipes* | *C.brevipes* | | yellow cups | herb | native |
| MNP | *Camissonia* | *claviformis* | *C.claviformis* | | brown-eyed primrose | herb | native |
| MNP | *Chaenactis* | *fremontii* | *C.fremontii* | | Fremont's pincushion | herb | native |
| MNP | *Cryptantha* | *intermedia* | *C.intermedia* | | common cryptantha | herb | native |
| MNP | *Caulanthus* | *lasiophyllus* | *C.lasiophyllus* | | California mustard | herb | native |
| MNP | *Chorizanthe* | *rigida* | *C.rigida* |  | Devil's spineflower | herb | native |
| MNP | *Erodium* | *cicutarium* | *E.cicutarium* | | filaree | herb | exotic |
| MNP | *Eriastrum* | *eremicum* | *E.eremicum* | | desert woolystar | herb | native |
| MNP | *Eremalche* | *exilis* | *E.exilis* |  | white mallow | herb | native |
| MNP | *Eschscholzia* | *minutiflora* | *E.minutiflora* | | coville poppy | herb | native |
| MNP | *Emmenanthe* | *penduliflora* | *E.penduliflora* | *penduliflora* | whispering bells | herb | native |
| MNP | *Eriophyllum* | *wallacei* | *E.wallacei* | | woolly daisy | herb | native |
| MNP | *Langloisia* | *setosissima* | *L.setosissima* | | bristly langlosia | herb | native |
| MNP | *Monoptilon* | *bellioides* | *M.bellioides* | | desert star | herb | native |
| MNP | *Malacothrix* | *glabrata* | *M.glabrata* | | desert dandelion | herb | native |
| MNP | *Nama* | *demissum* | *N.demissum* | | purple mat | herb | native |
| MNP | *Phacelia* | *crenulata* | *P.crenulata* | | notch-leaf phacelia | herb | native |
| MNP | *Pholistoma* | *membranaceum* | *P.membranaceum* | | white fiesta flower | herb | native |
| MNP | *Plantago* | *ovata* | *P.ovata* | *insularis* | desert plantain | herb | native |
| MNP | *Rafinesquia* | *neomexicana* | *R.neomexicana* | | desert chicory | herb | native |
| MNP | *Salvia* | *columbariae* | *S.columbariae* | | desert chia | herb | native |
| MNP | *Schismus* | *barbatus* | *S.barbatus* | | kelch grass | grass | exotic |
| PAN | *Aristida* | *adscensionis* | *A.adscensionis* | | sixweeks treeawn | grass | native |
| PAN | *Amsinckia* | *grandiflora* | *A.grandiflora* | | dandelion | herb | native |
| PAN | *Aastragalus* | *lentiginosus* | *A.lentiginosus* | | spotted locoweed | herb | exotic |
| PAN | *Acmispon* | *wrangelianus* | *A.wrangelianus* | | Chilean bird's-foot trefoil | herb | native |
| PAN | *Bromus* | *hordeaceus* | *B.hordeaceus* | | soft brome | grass | exotic |
| PAN | *Bromus* | *hordeaceus* | *B.hordeaceus* | | soft brome | grass | exotic |
| PAN | *Cryptantha* | *barbigera* | *C.barbigera* | | bearded cryptantha | herb | native |
| PAN | *Castilleja* | *exserta* | *C.exserta* |  | owls clover | herb | native |
| PAN | *Cryptantha* | *intermedia* | *C.intermedia* | | common cryptantha | herb | native |
| PAN | *Caulanthus* | *lasiophyllus* | *C.lasiophyllus* | | California mustard | herb | native |
| PAN | *Dichelostemma* | *capitatum* | *D.capitatum* | | blue dicks | herb | native |
| PAN | *Deinandra* | *kelloggii* | *D.kelloggii* | | tarweed | herb | native |
| PAN | *Erodium* | *cicutarium* | *E.cicutarium* | | filaree | herb | exotic |
| PAN | *Hordeum* | *vulgare* | *H.vulgare* |  | barley | grass | exotic |
| PAN | *Lasthenia* | *gracilis* | *L.gracilis* |  | gold fields | herb | native |
| PAN | *Phacelia* | *tanacetifolia* | *P.tanacetifolia* | | notch-leaf phacelia | herb | native |
| PAN | *Plantago* | *ovata* | *P.ovata* | *insularis* | desert plantain | herb | native |
| PAN | *Salvia* | *columbariae* | *S.columbariae* |  | desert chia | herb | native |
| PAN | *Schismus* | *barbatus* | *S.barbatus* | | kelch grass | grass | exotic |
