# Supplementary material for: Shrubs indirectly increase desert seedbanks through facilitation of the plant community
Source: PLoS One. 2019 Apr 24;14(4):e0215988. doi: 10.1371/journal.pone.0215988 (PMC6481865; doi:10.1371/journal.pone.0215988)
Supplement: S3 Appendix — (DOCX) [file pone.0215988.s003.docx]

**Supplementary 3 – Images of treatment setup**

**
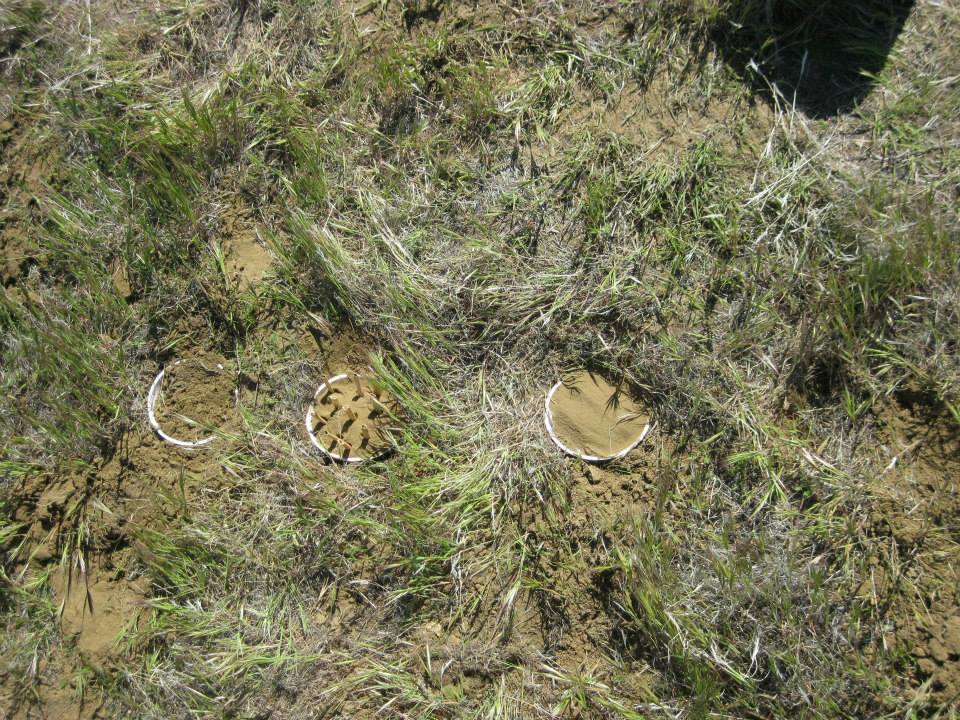
**

**Figure A:** Treatment plots at PAN in an open microsite in the following order: -P+S, +P-S (artificial plants), -P-S, and +P+S (control).

**
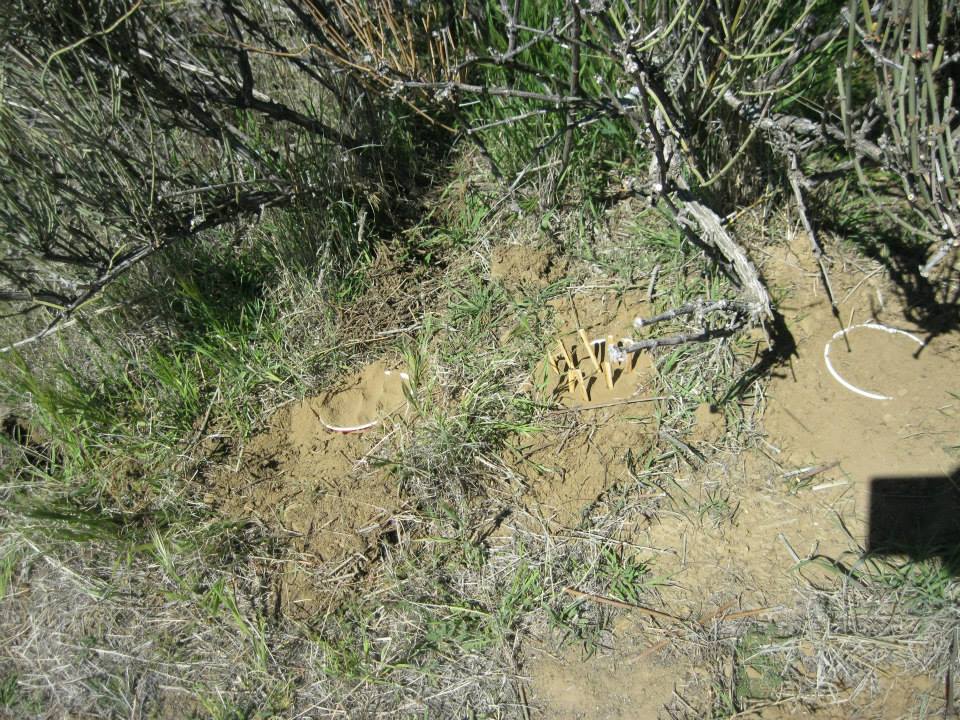
**

**Figure C:** Treatment plots at PAN in a shrub microsite in the following order: +P+S (control), -P-S, +P-S (artificial plants), and -P+S.
