# Supplementary material for: Shrubs indirectly increase desert seedbanks through facilitation of the plant community
Source: PLoS One. 2019 Apr 24;14(4):e0215988. doi: 10.1371/journal.pone.0215988 (PMC6481865; doi:10.1371/journal.pone.0215988)
Supplement: S4 Appendix — (DOCX) [file pone.0215988.s004.docx]

**Table A:** The different treatments used within the experiment

| **Treatment** | **Plants** | **Seedbank** | **Description** |
| --- | --- | --- | --- |
| +P+S | Ambient plant community | Ambient seedbank (persistent) | Positive control |
| -P+S | Plant community removed | Ambient seedbank (persistent) | Seedbank intact, plants removed |
| +P-S | Ambient plant community replaced with artificial plants | Ambient seedbank removed | Seedbank removed, plants replaced with mimics |
| -P-S | Plant community removed | Ambient seedbank removed | Negative control |
