# Supplementary figures and images for: Shrubs indirectly increase desert seedbanks through facilitation of the plant community
Source: PLoS One. 2019 Apr 24;14(4):e0215988. doi: 10.1371/journal.pone.0215988 (PMC6481865; doi:10.1371/journal.pone.0215988)

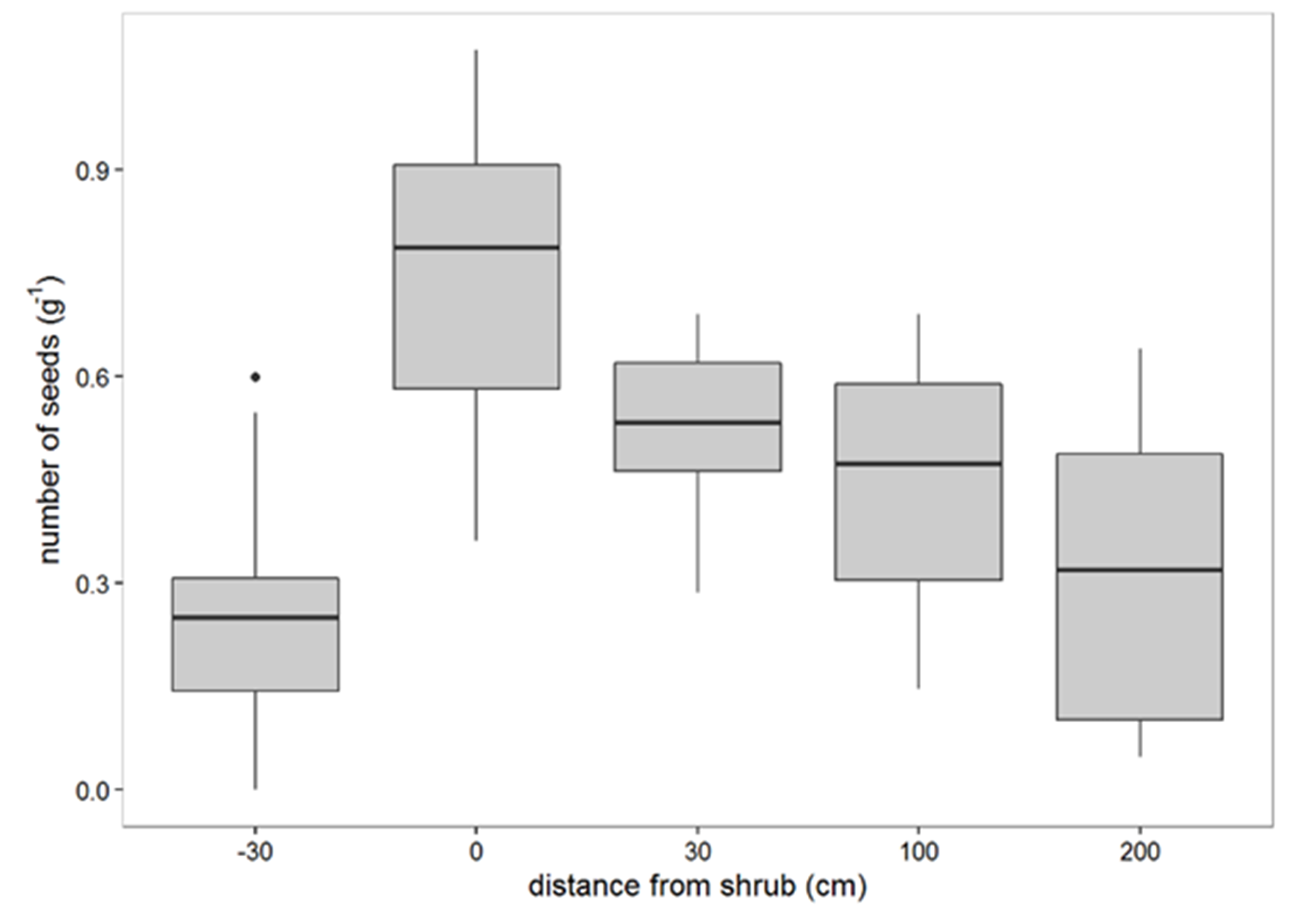

Supplement: S1 Fig — (TIF) [file pone.0215988.s005.tif]

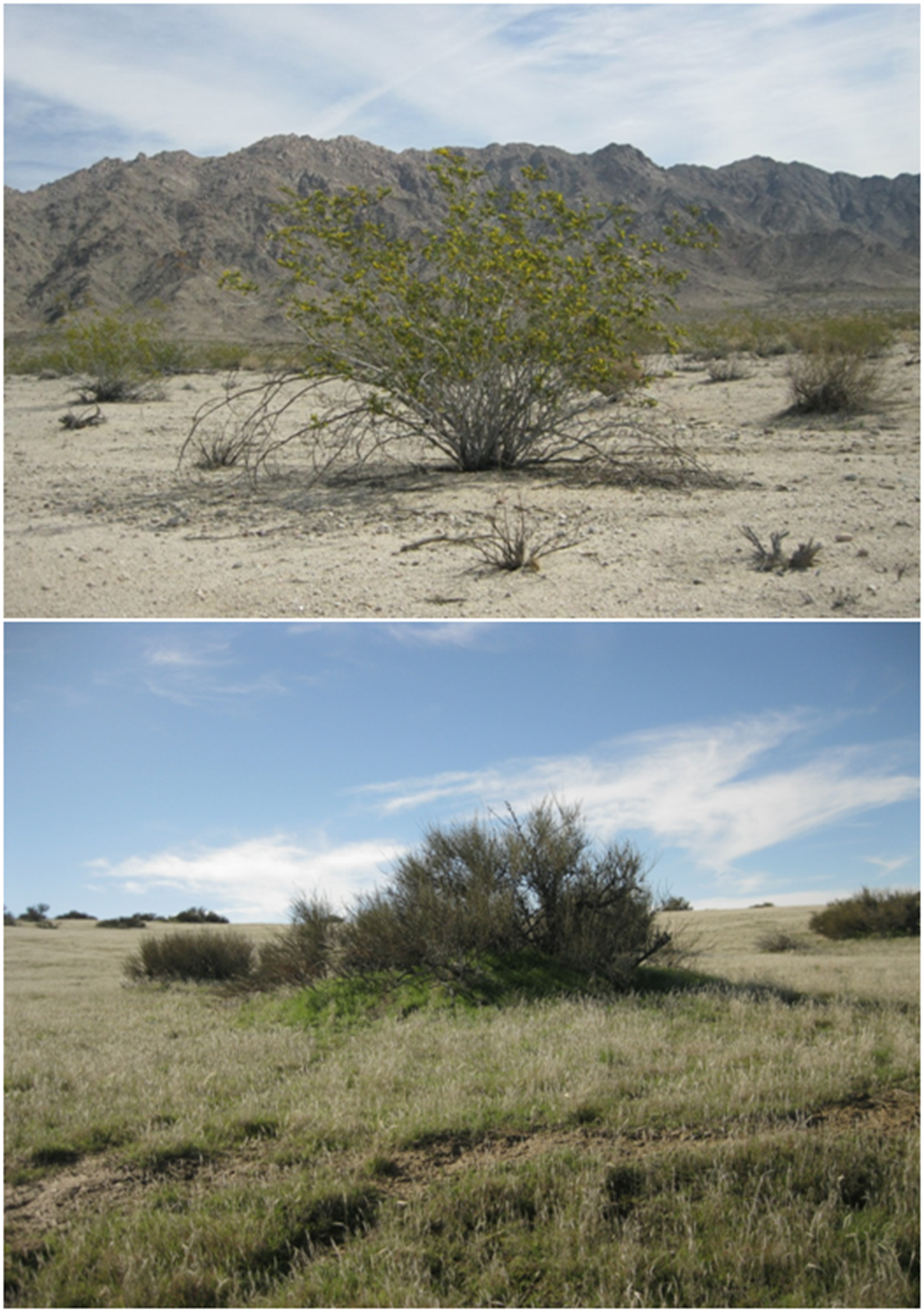

Supplement: S2 Fig — (TIF) [file pone.0215988.s006.tif]
